# Sodium cantharidate promotes autophagy in breast cancer cells by inhibiting the PI3K–Akt–mTOR signaling pathway

**DOI:** 10.3389/fphar.2022.1000377

**Published:** 2022-11-02

**Authors:** Jin-Long Pang, Lian-Song Xu, Qian Zhao, Wen-Wen Niu, Xiang-Yu Rong, Shan-Shan Li, Xian Li

**Affiliations:** ^1^ Anhui Engineering Technology Research Center of Biochemical Pharmaceutical, Bengbu, Anhui, China; ^2^ School of Pharmacy, Bengbu Medical College, Bengbu, Anhui, China; ^3^ New Technologies for Chinese Medicine Drinker Manufacturing Anhui Provincial Key Laboratory, Hefei, Anhui, China; ^4^ Postdoctoral Workstation of Anhui Xiehecheng Drinker Tablets Co.,Ltd., Bozhou, Anhui, China

**Keywords:** sodium cantharidate, PI3K- Akt-mTOR pathway, breast cancer, autophagy, apoptosis

## Abstract

Sodium cantharidate (SCA) is a derivative of cantharidin obtained by its reaction with alkali. Studies have shown that it inhibits the occurrence and progression of several cancers. However, therapeutic effects of SCA on breast cancer are less well studied. This study aimed to clarify the effect of SCA on breast cancer cells and its mechanism, and to provide a scientific basis for the clinical use of SCA for the treatment of breast cancer. The results of cell counting kit-8, colony formation assay, and 5-ethynyl-2′-deoxyuridine staining showed that SCA inhibited breast cancer cell proliferation. Wound-healing and transwell assays demonstrated that SCA inhibited the migration and invasion of breast cancer cells. Transmission electron microscopy revealed that SCA induced autophagy in breast cancer cells. RNA sequencing technology showed that SCA significantly regulated the phosphoinositide 3-kinase–Akt–mammalian target of rapamycin (PI3K–Akt–mTOR) pathway, which was further verified using western blotting. The inducing effect of SCA on breast cancer autophagy was reversed by the mTOR activator MHY1485. In addition, subcutaneous xenograft experiments confirmed that SCA significantly inhibited tumor growth *in vivo*. Hematoxylin-eosin, TdT-mediated dUTP nick-end labeling, and immunohistochemical staining indicated that SCA induced tumor cell autophagy and apoptosis in nude mice without causing organ damage. In summary, we found that SCA promoted breast cancer cell apoptosis by inhibiting the PI3K–Akt–mTOR pathway and inducing autophagy.

## Graphical Abstract

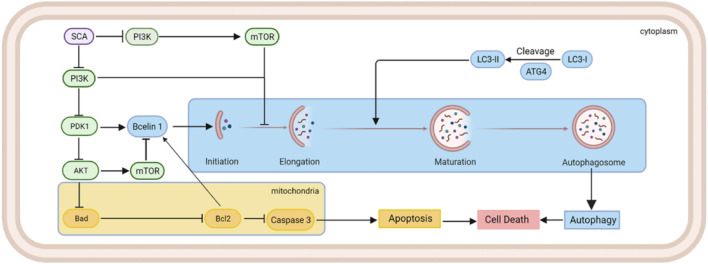

## 1 Introduction

Female breast cancer ranks at the top of cancer incidence worldwide, seriously threatening women’s health (Sung et al., 2021). Breast cancer cells exhibit highly divergent gene expression profiles, and different breast cancer subtypes show substantial differences in biological behavior, therapeutic efficacy, and prognosis (Prat et al., 2015). The treatment of breast cancer has evolved rapidly over the past decades, and a five-year survival rate of more than 90% has been achieved. However, because of high intratumoral heterogeneity, recurrence following treatment, metastasis, and drug resistance still pose a great challenge in breast cancer treatment (Beca et al., 2016; Allemani et al., 2018). Therefore, further development of new therapeutic agents is required to provide effective strategies for breast cancer treatment to improve therapeutic outcomes.

Autophagy is an intracellular clearance process that is critically involved in metabolic diseases, inflammation, degenerative diseases, and tumorigenesis (De et al., 2016). Autophagy occurs when intracellular components, including organelles, are engulfed by autophagosomes and subsequently fuse with lysosomes to digest the functional luminal material (Wu et al., 2021). Autophagy plays multiple roles in cancer, and its specific role largely depends on the stage of cancer development. On the one hand, autophagy can clean up unnecessary materials inside cells to maintain the intracellular environment and genomic stability and inhibit tumorigenesis. However, moderate autophagy can activate the recycling machinery and utilize intracellular resources, improving the viability of cancer cells under stress conditions (Ulasov et al., 2019). In tumor cells, autophagy is mainly regulated by the AMP-activated protein kinase (AMPK) and mammalian target of rapamycin (mTOR) pathways. As an energy sensor, AMPK can be activated under conditions of cellular energy stress. Activated AMPK phosphorylates Unc-51-like kinase (ULK1) and the tuberous sclerosis TSC1/TSC2 complex, thereby inhibiting mTOR complex 1 (mTORC1) activation to induce autophagy (Gwinn et al., 2008; Egan et al., 2011). An essential component of the mTORC1 complex, mTOR kinase, phosphorylates and inactivates the autophagy regulatory complex (formed by ULK1 and its interacting proteins ATG13, FIP200, and ATG101), thereby affecting autophagosome biogenesis (Feldman et al., 2009; Marinkovic et al., 2018). Although the role of autophagy in breast cancer has not been clearly established, initial reports suggest that inactivation of autophagy genes are associated with breast cancer initiation and progression (Qu et al., 2003; Yue et al., 2003). Studies have shown that during breast cancer development, phosphoinositide 3-kinase (PI3K), phosphate and tension homology deleted on chromosome ten (PTEN), and p53 play important roles, and PI3K–Akt–mTOR and PTEN pathways are the most important therapeutic targets in breast cancer treatment (McKenna et al., 2018). Notably, abnormalities in the PI3K–Akt–mTOR pathway also play a critical role in the development of breast cancer drug resistance, suggesting that this pathway is a highly promising candidate in breast cancer therapy.

Sodium cantharidate (SCA; Guizhou Shenqi Pharmaceutical Co., Ltd., Qiannan, China) is a compound extracted from the dried body of Meloidae insects (Deng et al., 2017). Studies have shown that SCA can induce hepatoma cell apoptosis by triggering endoplasmic reticulum stress (Ye et al., 2019). Furthermore, SCA can induce HepG2 cell apoptosis *via* the LC3 autophagy pathway (Tao et al., 2017). Moreover, in pancreatic cancer cells, SCA exerts antitumor effects by activating p53 (Liu et al., 2021). However, the application of SCA for breast cancer treatment has not yet been reported.

This study aimed to elucidate the therapeutic effect of SCA on breast cancer and the underlying mechanism. *In vivo*, SCA inhibited tumor growth in nude mice. *In vitro*, SCA induced autophagy in breast cancer cells *via* the PI3K–Akt–mTOR pathway and decreased the Bcl-2/Bax ratio to induce apoptosis in breast cancer cells. In conclusion, our study provides an effective therapeutic strategy for breast cancer.

## 2 Materials and methods

### 2.1 Cell lines and cell culture

The MCF-7 cell line was purchased from Procell (Wuhan, China) and cultured in high-glucose Dulbecco’s modified Eagle’s medium (Gibco, New York, NY, United States) containing 12% fetal bovine serum (ExCell, Shanghai, China) and 1% antibiotics (Biosharp, Hefei, China). The MDA-MB-231-Luc cell line was purchased from Zhong Qiao Xin Zhou Biotechnology Co., Ltd. (Shanghai, China) and cultured in Dulbecco’s modified Eagle’s medium containing puromycin (2 μg/ml). The incubator conditions were 37°C, saturated humidity, and 5% CO_2_.

### 2.2 Cell counting kit-8 assay

Cells were seeded into 96-well plates (Biosharp) and treated with SCA (0, 4, 8, 16, 32, and 64 μmol/L) for 24, 48, and 72 h when the cell confluence reached 60%. Then, fresh medium containing CCK-8 (100 µL/ml; APExBIO, Houston, TX, United States) was added and incubated for 1 h at 37°C. The absorbance was measured at a wavelength of 450 nm using a microplate reader (BioTek Instruments, Vermont, United States), and the cell viability was calculated.

### 2.3 Colony formation assay

Cells were seeded into 6-well plates (Biosharp) and treated with SCA (0, 1, 2, and 4 μmol/L) after the cells had fully attached. Incubation was continued for 12 days in the incubator. Subsequently, the cells were fixed using 4% paraformaldehyde (Biosharp) for 15 min, washed with phosphate-buffered saline (PBS, Biosharp), and stained with crystal violet (Biosharp) for 30 min. Clonogenicity was observed and photographed with inverted microscope (Leica Dmil, Weztlar, Germany).

### 2.4 Invasion and migration assays

In the invasion experiment, Matrigel (Corning, New York, United States) and PBS were mixed at a ratio of 1:8 and added to the upper chamber of the transwell chamber (Corning). Then, Dulbecco’s modified Eagle’s medium without serum was added and cells were seeded in the upper chamber (1 × 10^4^/well). In the migration experiment, cells were directly seeded without Matrigel in the lower chamber. After continued incubation for 24 h, cells were fixed using 4% paraformaldehyde for 5 min and then stained with crystal violet staining solution for 10 min. Then, the invading and migrating cells were calculated.

### 2.5 Wound-healing assay

Cells were seeded in 6-well plates (5 × 10^5^/well) and when a cell confluence of 90% was reached, a pipette was used to create a wound by drawing a straight line in the bottom of the 6-well plates. The cells were then photographed with inverted microscope. Subsequently, the cells were treated with SCA (0, 1, 2, and 4 μmol/L) and photographed after culturing for 24 h. The invasion distance was then calculated.

### 2.6 5-Ethynyl-2′-deoxyuridine staining

Cells were seeded in 6-well plates (2 × 10^5^/well) and treated with SCA (0, 4, 8, and 16 μmol/L) for 24 h. Subsequently, the cells were treated with EdU (10 μmol/L; Beyotime, Shanghai, China), incubated for 3 h, fixed with crystal violet, and treated with 0.3% Triton X-100 for 15 min (Beyotime) to increase membrane permeability. This was continued for 30 min in the dark using a click reaction mixture. Finally, the cells were incubated with 4′,6-diamidino-2-phenylindole (DAPI) (Beyotime) for 10 min. Stained cells were visualized using a live cell workstation (ZEISS, Oberkochen, Germany).

### 2.7 Western blot assay

Treated cells were lysed using a lysis buffer (Biosharp) and centrifuged (4°C, 12000 × *g*, 20 min). The supernatant was collected and the protein concentration was calculated using a protein quantification kit (Biosharp). The protein samples were subsequently boiled (95°C, 5 min) and then cooled down. A sample containing 60 µg of protein was added to each well for electrophoresis (80 V, 30 min; 120 V, 2 h). Subsequently, proteins were transferred onto a membrane (Millipore, MA, United States; 200 mA). The membranes were blocked in 5% non-fat milk (Biosharp) for 2.5 h, washed three times with TPBS (PBS contains 0.1% Tween 20), and incubated with primary antibodies (Ki67: Proteintech, #27309-1-AP, Wuhan, China; Bcl-2:CST, Boston, MA, United States, #15071T; Bax:CST, #5023T; p62:CST, #8025T; LC3-2:CST, #4108S; Beclin1:CST, #3495T; PI3K: CST, #4249T; AKT: CST, #4691T; mTOR: CST, #2983T; p-PI3K: CST, #17366S; p-AKT: CST, #4060S; p-mTOR: CST, #5536S) overnight at 4°C. The membranes were then washed and co-incubated with the corresponding secondary antibodies (Proteintech, #SA00001-1, SA00001-2, Wuhan, China) for 2 h at 20°C, and the protein bands were exposed using the ECL developing fluid (Millipore).

### 2.8 Flow cytometry

Cells were seeded in 6-well plates (2 × 10^5^/well) and treated with SCA (0, 4, 8, and 16 μmol/L) for 24 h. Then, the cells were collected and centrifuged (2500 × *g*, 10 min). Then, 300 μl binding solution was added to resuspend the cells, 5 μl FITC reagent (Bestbio, Shanghai, China) was added in the dark for 15 min, and 3 μl PI reagent (Bestbio, Shanghai, China) was added for 10 min. Flow cytometry was then performed to assess apoptosis in each group.

### 2.9 RNA sequence

Cells were treated with SCA (10 μmol/L) for 24 h, collected in a cryopreservation tube (Biosharp), stored at −80°C, and analyzed by Lianchuan Biotechnology Co., Ltd.

#### 2.9.1 mRNA sequencing

First, RNA was extracted using TRIzol (Invitrogen, CA, United States), purified, and verified using agarose gel electrophoresis. Polyadenylic acid mRNA was captured using magnetic beads (Dynabeads Oligo (dT), Thermo Fisher Scientific, United States) and fragmented under high-temperature conditions. Fragmented RNA was used for cDNA synthesis. Then, the complex double-strands of DNA and RNA were converted into double-stranded DNA. Magnetic beads were then used to screen, purify, and amplify the size of the fragments. Finally, we used Illumina Novaseq 6000 (LC Bio Technology Co., Ltd. Hangzhou, China) to perform paired-end sequencing of the fragments using the sequencing mode PE150.

#### 2.9.2 Sequence and primary analyses

First, cutadapt (https://cutadapt.readthedocs.io/en/stable/) was used to remove the reads that contained adaptor contamination, and low-quality and repetitive sequences were removed to obtain CleanData. HISAT2 (https://daehwankimlab.github.io/hisat2/) was used to compare the CleanData to the human genome (Homo sapiens, Ensembl v96), and then StringTie (http://ccb.jhu.edu/software/stringtie) was used to assemble and merge genes. The gffcompare (http://ccb.jhu.edu/software/stringtie/gffcompare.shtml) software was used to compare the transcript and the reference annotation to get the final annotation result. DESeq2 (http://www.bioconductor.org/packages/release/bioc/html/DESeq2.html) was used to analyze significant differences between samples. Subsequently, gene ontology and Kyoto Encyclopedia of Genes and Genomes enrichment analyses were performed using Blast2GO and KOALA (KEGG Orthology And Links Annotation).

### 2.10 Transmission electron microscopy

Cells were treated with SCA (10 μmol/L) for 24 h and then fixed with 2.5% glutaraldehyde (Servicebio, Wuhan, China) overnight, followed by ethanol dehydration. Then, resin was used to infiltrate and embed the cells, and a low-temperature ultraviolet polymerizer was used to polymerize the resin at −20°C for more than 48 h. The resin blocks were then sectioned to a 70–80 nm thickness. The tissues were dyed in a 2% uranyl acetate saturated alcohol solution for 8 min, washed with 70% alcohol and three times with ultrapure water, and then dried. The images were obtained using TEM (HITACHI, Chiyoda-ku, Tokyo, Japan) by Servicebio.

### 2.11 Caspase3 activity detection

Cells were treated with SCA (0, 4, 8, and 16 μmol/L) for 24 h when the cell confluence reached 60%. Then, the cells were collected and 100 µl of lysis buffer was added per 2 million cells, lysed in an ice bath, and centrifuged (4°C, 12000 × *g*, 15 min). Then, 40 µl detection buffer was added to the 96-well plate. Next, 50 µl of the sample to be tested was added and properly mixed. The mixture was co-incubated with Ac-DEVD-pNA (37°C, 2 h; Ruixin Biotechnology Co., Ltd.,Quanzhou, China). The optical density value was determined at a wavelength of 405 nm. The absorbance of the pNA catalyzed by caspase3 in the sample was determined by subtracting the A_405_ of the blank control to the A_405_ of the sample.

### 2.12 Cyto-ID staining

Cells were treated with SCA (0, 4, 8, and 16 μmol/L) in 6-well plates for 24 h and then stained with Cyto-ID (37°C, 30 min; Enzo, New York, NY, United States). Excess dye was removed and the cells were stained with DAPI for 15 min and imaged using a live cell workstation (ZEISS). The above experiments were conducted in the dark.

### 2.13 Experiments in tumor-bearing nude mice

BALB/c nude mice were purchased from Shanghai Laboratory Animal Center (Shanghai, China) and cultured in a sterile environment. After a week of acclimatization, MDA-MB-231-Luc cells were inoculated into nude mice at a density of 5 × 10^6^/mouse. The control group was treated with saline, while the experimental group received a daily intraperitoneal injection of SCA (0.3 mg/kg). One week after the tumor cell injection, the signal intensity of the tumor was detected using the IVIS Imaging System (Bruker, Madison, WI, United States), and treatment was initiated. Twenty-one days later, the tumor signal intensity of the nude mice was recorded again. The tumor, liver, spleen, lung, and kidney were removed, and the tumor weight was measured. Hematoxylin-eosin staining and TdT-mediated dUTP nick-end labeling were performed and the tissues were imaged.

### 2.14 Molecular docking

The AutoDock Vina 1.1.21 (Trott et al., 2010) program was used to predict the binding mode of SCA to PI3K. The crystal structure of PI3K was obtained from the Protein Date Bank (PDB ID: 1E8X). Before docking, the crystal structure was prepared using PyMOL 2.5 to remove water molecules and ligands and to add hydrogen atoms. Then, a grid box size of 25 × 25 × 25 Å (Morris et al., 2009), which contained the entire active binding site, was defined. The prepared protein and compound structures were converted to a PDBQT format using AutoDock Tools 1.5.63. During docking, the exhaustiveness of the global search was set to 20, the maximum number of binding modes to 40, and other parameters were kept at default. Finally, the best docking pose was visually analyzed using PyMOL 2.5 and Maestro of academic edition.

### 2.15 Statistical analyses

Data were analyzed by one-way ANOVA and are presented as mean ± standard deviation (SD). The experimental data contained at least three repeated and independent experiments, and *p* < 0.05 indicated that the differences were statistically significant.

## 3 Results

### 3.1 SCA inhibits the activity of breast cancer cells

**FIGURE 1:**
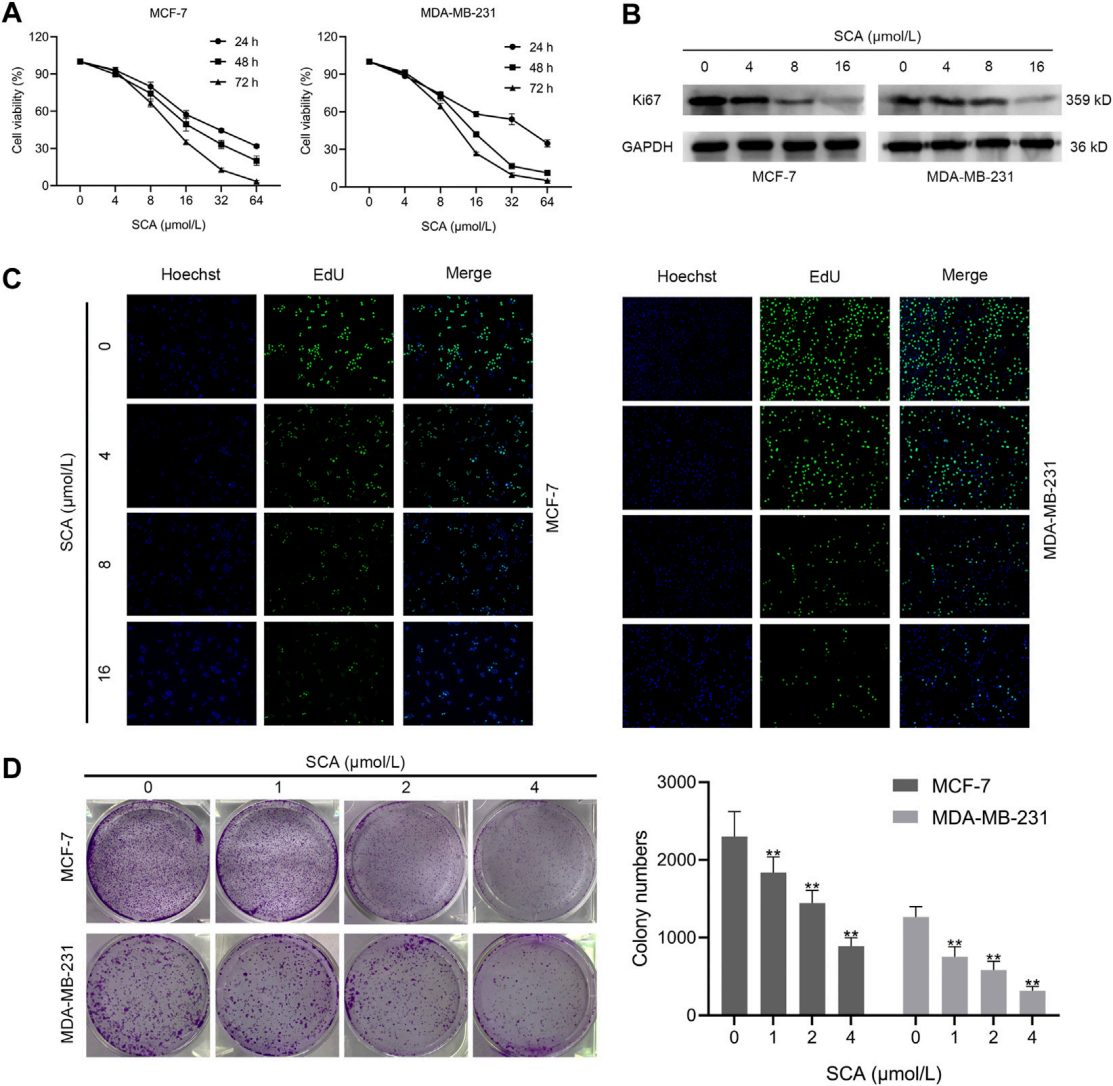
Sodium cantharidate (SCA) inhibits the activity of breast cancer cells. **(A)** CCK-8 was used to detect the viability of breast cancer cells after treatment with SCA at different concentrations and duration. **(B)** Western blot analysis of Ki67 levels in SCA-treated breast cancer cells for 24 h. **(C)** Fluorescence microscopy images of 5-ethynyl-2′-deoxyuridine (EdU)-stained cells after SCA treatment for 24 h (400×), scale bar: 2 µm. **(D)** Effect of SCA treatment on the colony forming ability of breast cancer cells. Data are expressed as the mean ± SD (*p < 0.05, **p < 0.01 compared with the control group).

To investigate the effect of SCA on the proliferation of breast cancer cells, we performed CCK-8, EdU staining, and colony formation assays. CCK-8 assay of breast cancer cells treated with SCA showed that SCA decreased cell proliferation in a concentration- and time-dependent manner (Figure 1A). In addition, we examined the Ki67 level in breast cancer cells; as predicted, SCA treatment significantly decreased the Ki67 level in breast cancer cells (Figure 1B). Furthermore, EdU staining results confirmed that the proliferation of breast cancer cells was inhibited by SCA treatment (Figure 1C; Supplementary Material is the Western blotting). To measure the effect of SCA on the proliferation of breast cancer cells under low concentration conditions, we performed colony formation and migration assays. The colony formation ability of breast cancer cells was significantly inhibited under low concentration conditions (Figure 1D).

### 3.2 SCA inhibits breast cancer cell invasion and migration

**FIGURE 2:**
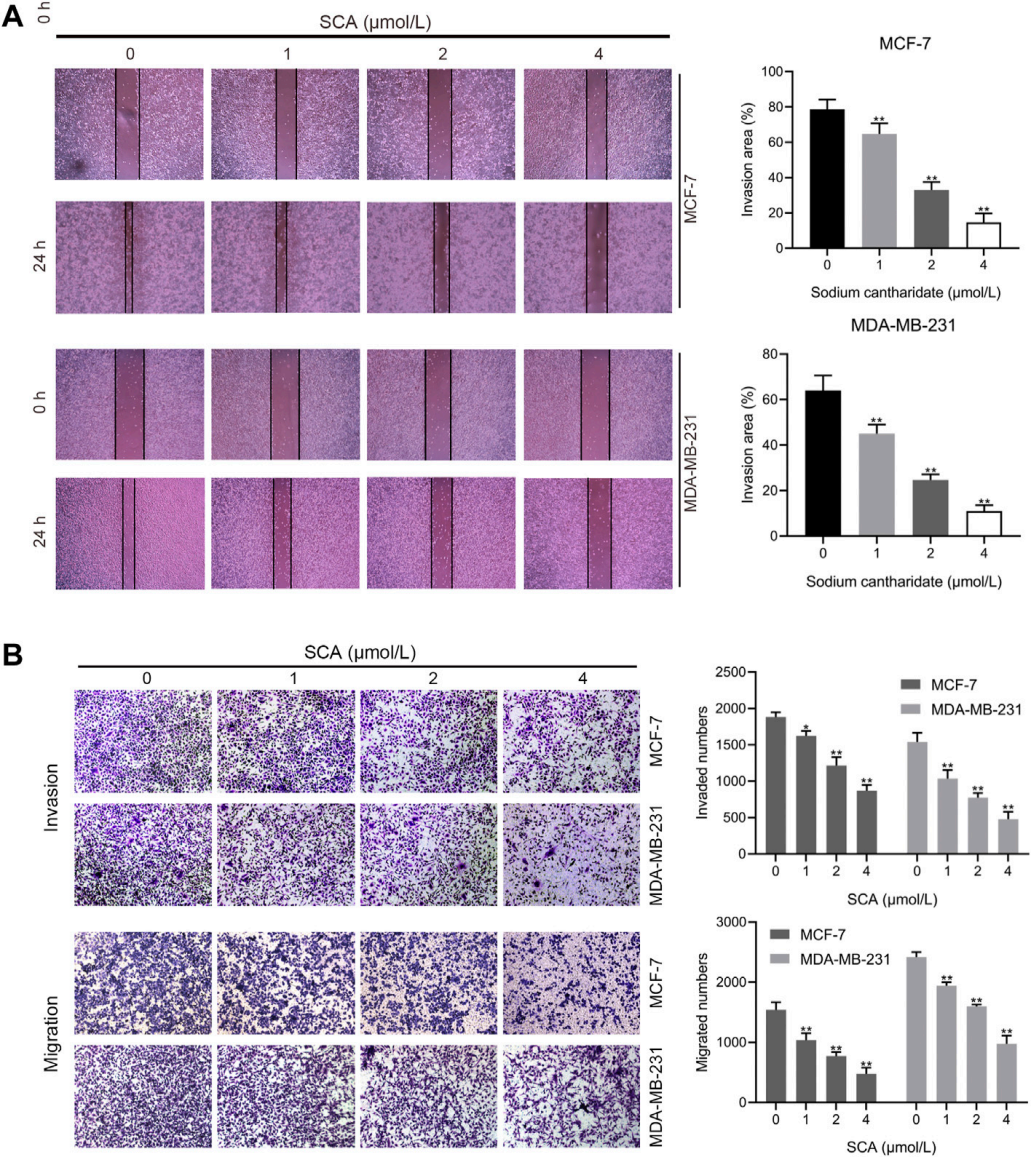
Sodium cantharidate (SCA) inhibits breast cancer cell invasion and migration. **(A,B)** Transwell assay showing the effect of SCA treatment on the invasive, migratory, and wound-healing abilities of breast cancer cells. Data are expressed as the mean ± SD (*p < 0.05, **p < 0.01 compared with the control group).

We examined the effects of SCA on breast cancer cell invasion and migration using transwell assays and wound-healing assays. SCA significantly inhibited breast cancer cell invasion and migration at low concentrations (Figure 2A) and reduced their healing ability (Figure 2B). These data showed that SCA can inhibit breast cancer cell proliferation, invasion, and migration, even at low concentrations.

### 3.3 SCA induces apoptosis in breast cancer cells

**FIGURE 3:**
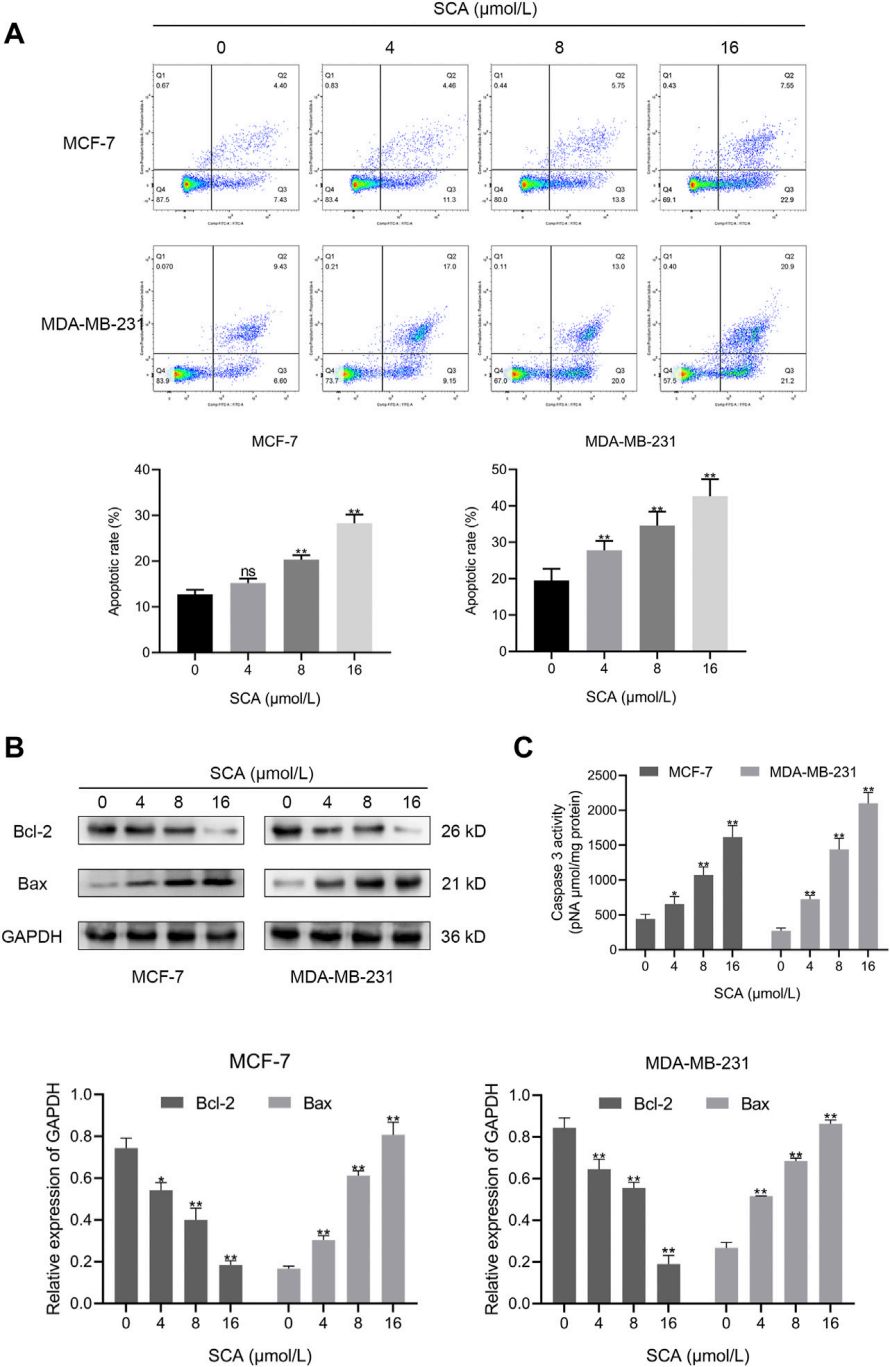
Sodium cantharidate (SCA) induces apoptosis in breast cancer cells. **(A)** The apoptosis rates of breast cancer cells treated with SCA for 24 h determined using flow cytometry. **(B)** Western blot analysis of apoptotic protein levels in SCA-treated breast cancer cells for 24 h. **(C)** Caspase3 activity in SCA-treated breast cancer cells after 24 h. Data are expressed as the mean ± SD (*p < 0.05, **p < 0.01 compared with the control group).

To observe the effect of SCA treatment on breast cancer cell apoptosis, we performed flow cytometry experiments. The results showed that SCA treatment increased the apoptosis rate of breast cancer cells in a concentration-dependent manner (Figure 3A). In addition, we detected the levels of Bcl-2 and Bax in MCF-7 and MDA-MB-231 cells. Consistent with the flow cytometry results, SCA treatment decreased the level of Bcl-2 and increased the level of Bax (Figure 3B Supplementary Material is the Western blotting). We additionally detected the activity of caspase3 and found that SCA treatment increased its activity in breast cancer cell lines (Figure 3C), indicating that SCA promoted breast cancer cell apoptosis in a dose-dependent manner.

### 3.4 SCA induces autophagy in breast cancer cells

**FIGURE 4:**
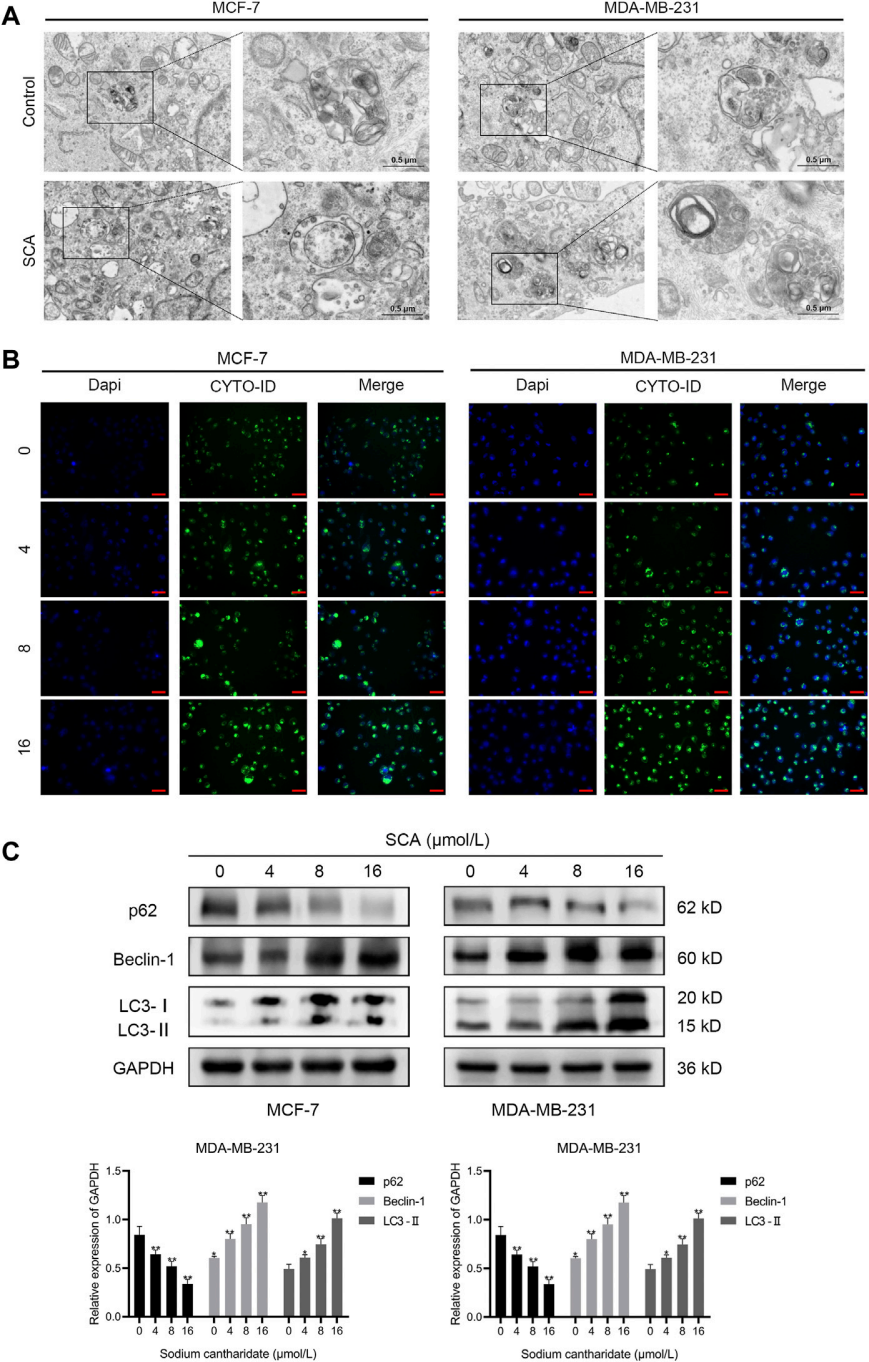
Sodium cantharidate (SCA) induces autophagy in breast cancer cells. **(A)** Transmission electron microscopy was performed to observe the effect of SCA on the submicroscopic structural morphology of breast cancer cells. **(B)** Fluorescence microscopy images of CYTO-ID staining in breast cancer cells after SCA treatment for 24 h (400×), scale bar: 2 µm. **(C)** Western blot analysis of the levels of autophagy-related proteins in breast cancer cells after SCA treatment for 24 h. Data are expressed as the mean ± SD (*p < 0.05, **p < 0.01 compared with the control group).

We used TEM to further define the manner in which SCA induces breast cancer cell death and found an increase in autophagosomes after SCA treatment in both cell lines (Figure 4A). To further define the occurrence of autophagy, we treated the two breast cancer cell lines with SCA and used an autophagy detection kit. In line with the TEM findings, we consistently detected stronger autophagy signals in the SCA-treated group (Figure 4B) than in the control group. Furthermore, we explored the effects of SCA treatment on autophagy-related proteins in the two cell lines, and found that the level of the autophagy protein p62 significantly decreased, whereas those of LC3-2 and Beclin1 significantly increased in the SCA-treated group compared to the levels in the control group, further confirming the occurrence of autophagy (Figure 4C Supplementary Material is the Western blotting). Therefore, we speculated that SCA treatment induced apoptosis in breast cancer cells by increasing the occurrence of autophagy.

### 3.5 The effect of SCA on breast cancer cells is related to the PI3K–Akt–mTOR signaling pathway

**FIGURE 5:**
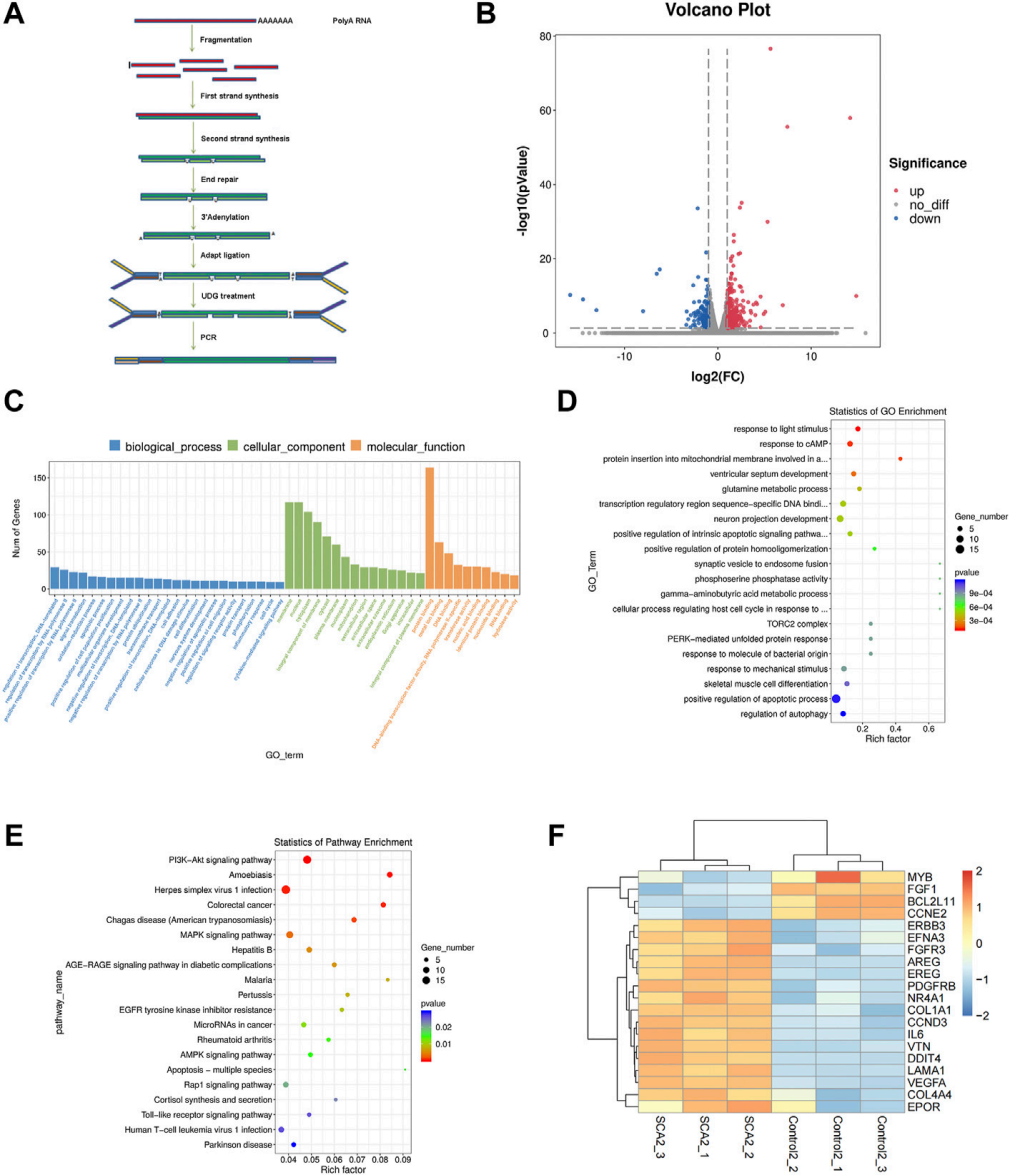
The effect of sodium cantharidate (SCA) on breast cancer cells is related to the PI3K–Akt–mTOR signaling pathway. **(A)** Experimental methods of mRNA sequencing analysis. **(B)** Volcano plot analysis of differentially expressed genes 24 h after SCA treatment. **(C,D)** Histogram and scatter plots of gene ontology enrichment of differentially expressed genes. **(E)** Kyoto Encyclopedia of Genes and Genomes pathway enrichment analysis of differentially expressed genes. **(F)** Heatmap analysis of differentially expressed genes. Red and blue colors indicate relative expression above and below the average, respectively.

To identify the mechanism of SCA-induced autophagy in breast cancer cells, we performed RNA sequencing on SCA-treated and untreated groups and identified dysregulated candidate genes, functions, and signaling pathways (Figures 5A–E). Among these genes, *ERBB3*, *FGFR3*, *AREG*, *FGF1*, and *DDIT4* are related to the PI3K–Akt–mTOR signaling pathway and *Bcl-2* is related to autophagy and apoptosis (Figure 5F; Supplementary Material is the raw data for RNA-Seq). Consistent with this, gene ontology analysis showed that SCA treatment affected autophagy in breast cancer cells, and Kyoto Encyclopedia of Genes and Genomes analysis showed that the PI3K–Akt–mTOR pathway was strongly affected (Figures 5D,E). Considerable evidence showed that the PI3K–Akt–mTOR signaling pathway is the main pathway involved in the intracellular regulation of autophagy. Taken together, the above data suggest that the SCA-induced regulation of autophagy in breast cancer cells is mediated by the PI3K–Akt–mTOR signaling pathway.

### 3.6 SCA induces autophagy in breast cancer cells *via* the PI3K–Akt–mTOR signaling pathway

**FIGURE 6:**
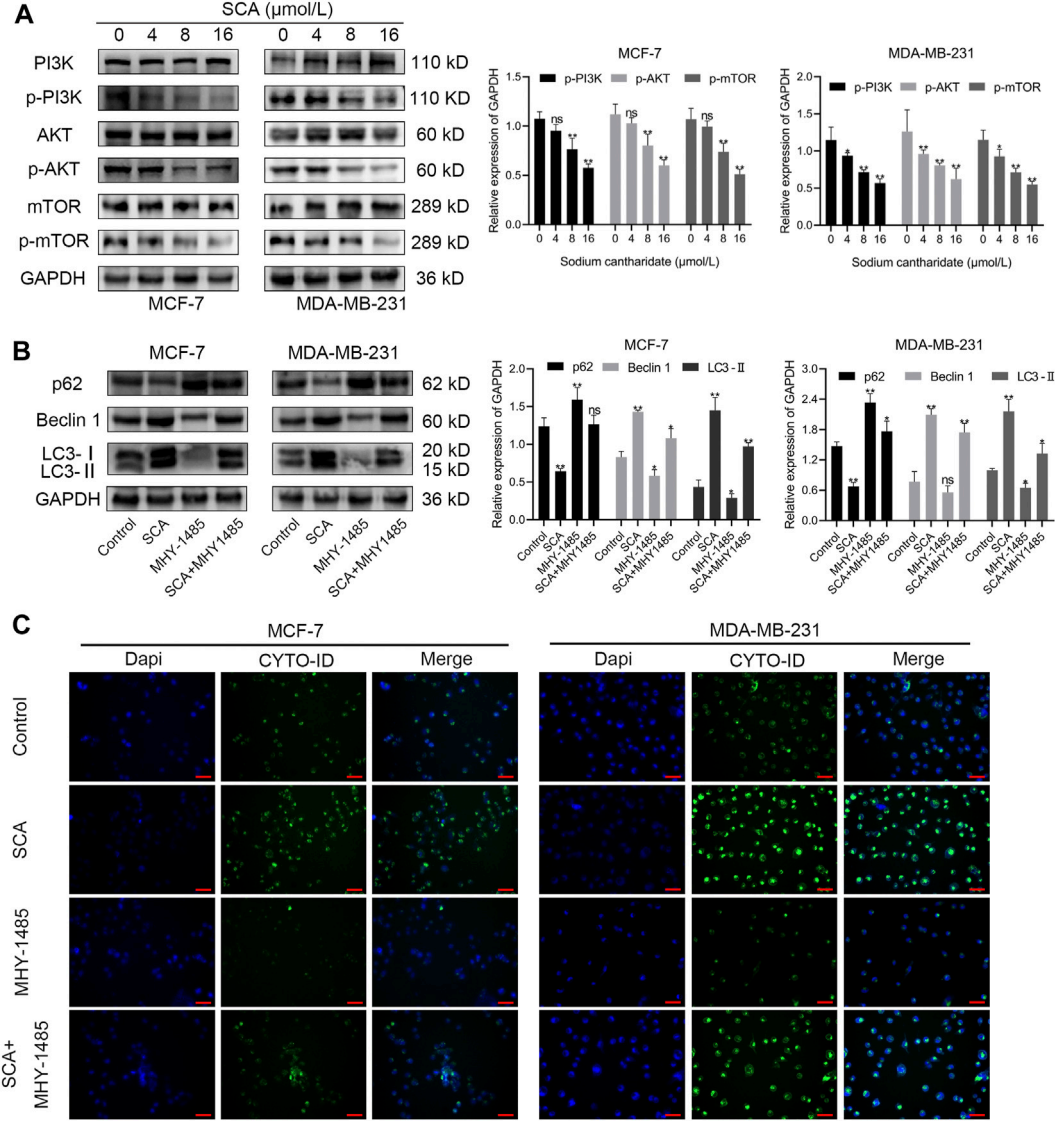
The effect of sodium cantharidate (SCA) on breast cancer cells is related to the PI3K–Akt–mTOR signaling pathway. **(A)** Western blot analysis of the levels of PI3K–AKT–mTOR pathway-related proteins in SCA-treated breast cancer cells for 24 h. **(B)** Western blot analysis of autophagy-related proteins affected by SCA and MHY1485 alone or in combination. **(C)** Fluorescence microscopy images of CYTO-ID-stained breast cancer cells after treatment with SCA and MHY1485 alone or in combination for 24 h (×400), scale bar: 2 µm. Data are expressed as the mean ± SD (*p < 0.05, **p < 0.01 compared with the control group).

The microarray results suggested that the autophagy inducing effect of SCA in breast cancer cells might be related to its regulation of the PI3K–Akt–mTOR signaling pathway. To test this, we first examined the effect of SCA on PI3K–Akt–mTOR signaling-related proteins in breast cancer cells. The results showed that SCA treatment decreased the p-PI3K, p-AKT, and p-mTOR levels (Figure 6A Supplementary Material is the Western blotting). To confirm that SCA-mediated autophagy was driven by mTOR signaling, we used the mTOR agonist MHY1485 in combination with SCA or alone to observe its effects on autophagy in breast cancer cells. We found that the SCA-induced autophagy was attenuated by MHY1485 (Figure 6B Supplementary Material is the Western blotting). To confirm the occurrence of autophagy, we used an autophagy kit; as expected, MHY1485 attenuated the SCA-induced autophagy in breast cancer cells (Figure 6C). These data illustrated that SCA activated autophagy in breast cancer cells by inhibiting the PI3K–Akt–mTOR signaling pathway, thereby inducing apoptosis.

### 3.7 SCA inhibits tumorigenesis in nude mice

**FIGURE 7:**
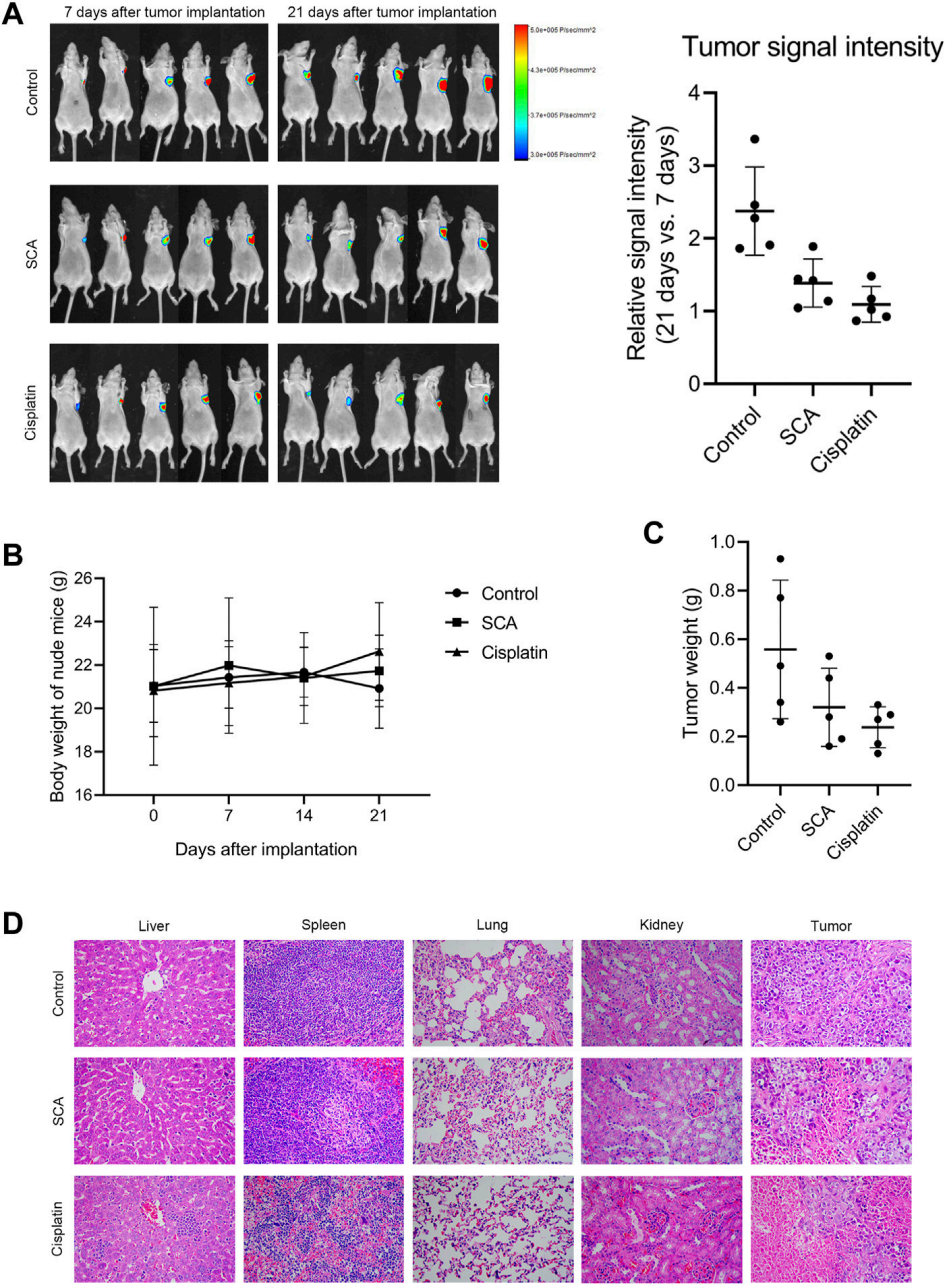
Sodium cantharidate (SCA) inhibits tumorigenesis in nude mice. **(A)** Tumor signal intensity before and after SCA treatment. **(B)** Body weight change curves of nude mice during SCA treatment. **(C)** Tumor weight after 21 days of treatment. **(D)** Hematoxylin-eosin staining images (400×) of the liver, spleen, lung, kidney, and tumor of nude mice after SCA treatment for 21 days, scale bar: 2 µm. Data are expressed as the mean ± SD (*p < 0.05, **p < 0.01 compared with the control group).

We used xenograft tumor models to study the effect of SCA on tumorigenesis in nude mice. We found that the SCA-treated mice exhibited a significantly lower tumor growth rate without showing significant effects on the body weight compared with that of the control group. The tumor weight of the SCA-treated nude mice was also lower than that of the control mice after the end of treatment (Figures 7A–C). To evaluate the toxic effects of SCA treatment, hematoxylin-eosin staining was also performed, which showed that SCA treatment caused no obvious damage to the liver, spleen, lung, and kidney.

### 3.8 SCA induces autophagy and apoptosis of breast cancer cells in nude mice

**FIGURE 8:**
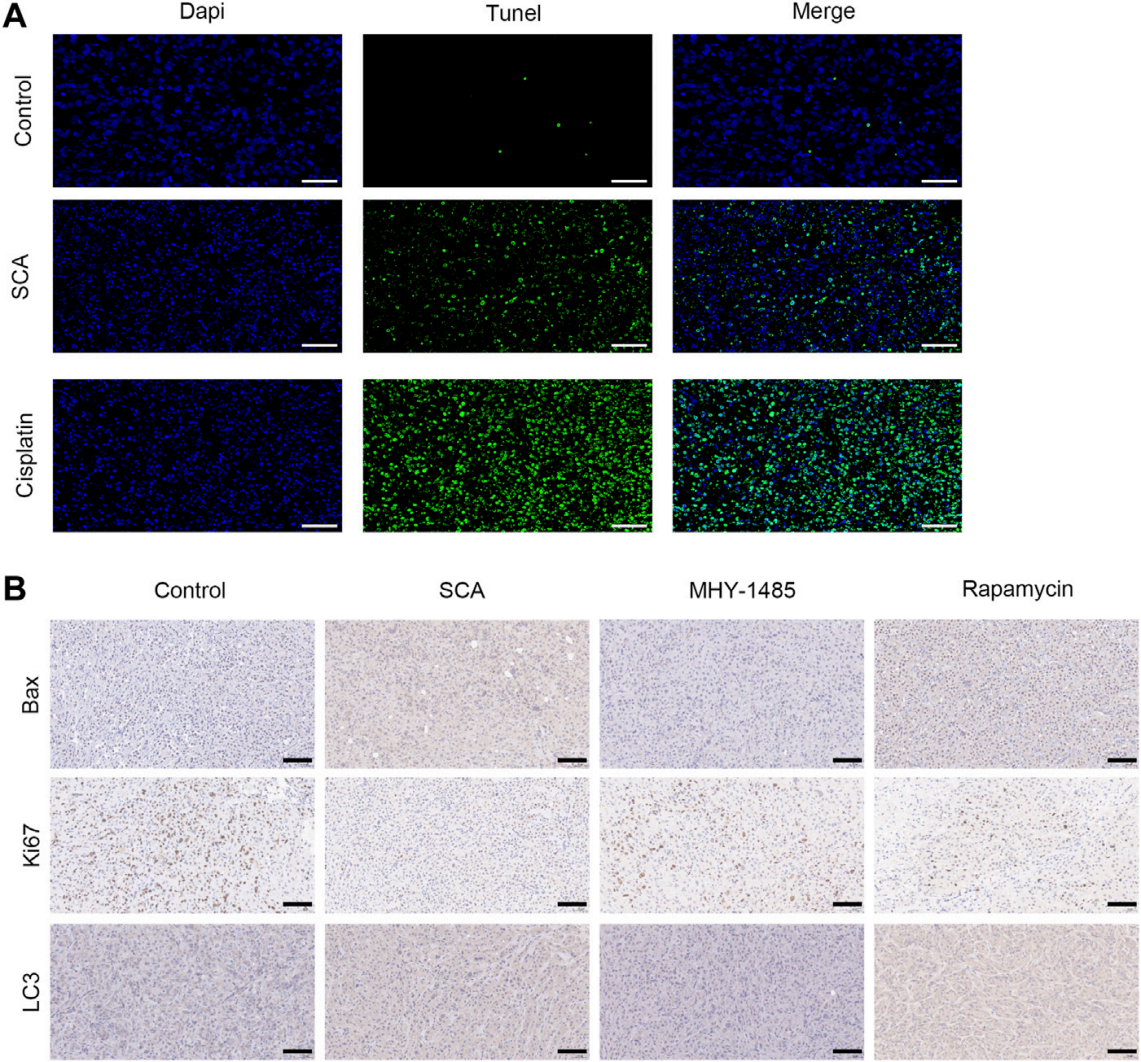
Sodium cantharidate (SCA) induces autophagy and apoptosis of breast cancer cells in nude mice. **(A)** Images of TdT-mediated dUTP nick-end labeling staining of tumors in each group after 21 days of treatment (400×), scale bar: 2 µm. **(B)** Caspase3 activity in tumor tissues after treatment with SCA for 21 days. Data are expressed as the mean ± SD (*p < 0.05, **p < 0.01 compared with the control group).

We detected apoptosis of nude mouse tumor tissue cells using TdT-mediated dUTP nick-end labeling staining (Figure 8A). Moreover, immunohistochemical staining showed that the Ki67, Bax, and LC3 levels in tumor tissues were decreased after treatment with SCA (Figure 8B). These results indicate that SCA induces autophagy and apoptosis of breast cancer cells in nude mice.

### 3.9 SCA directly binds and inhibits PI3K

**FIGURE 9:**
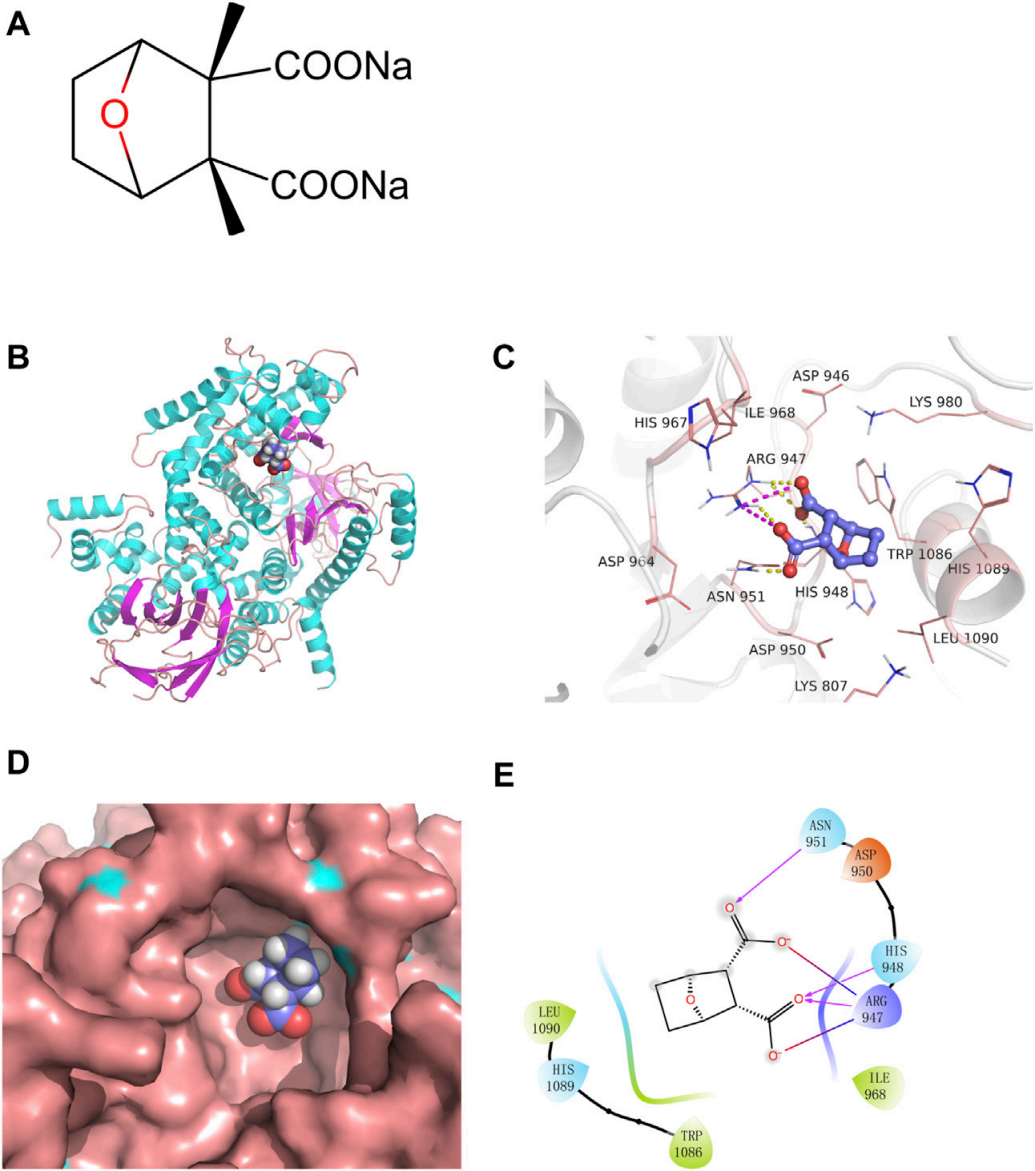
Sodium cantharidate (SCA) directly binds and inhibits PI3K. **(A)** Molecular structure of SCA. **(B)** The 3D structure of the SCA–PI3K complex. **(C)** Zoom-in view of SCA interactions with residues from the PI3K active site; yellow dashed lines represent hydrogen bonds and burgundy dashed lines represent salt bridges. **(D)** Zoom-in view of SCA bound to the active pocket of PI3K. **(E)** 2D representation of the interactions between SCA and PI3K; burgundy lines indicate hydrogen bonds, red-to-blue gradient lines represent salt bridges, and hydrophobic residues are colored in green.

Molecular docking is a convenient and effective way to explore the interaction of small molecules with targets. Here, the docking program Vina 1.1.2 was used to capture the binding affinity of SCA (Figure 9A) to PI3K.

The results showed that SCA binds to the active pocket (Figure 9B). Based on the surface binding mode, SCA fits well in the active cavity of PI3K (Figure 9D). Furthermore, we analyzed the binding mode of this complex (Figures 9C,E). SCA formed hydrogen bond interactions with Arg947, HisS948, and Asn950, and hydrophobic interactions with Trp1086 and Leu1090. Moreover, the two carboxyl groups of the compound formed two salt bridges with Arg947, which further strengthens the stability of the SCA–PI3K complex.

## 4 Discussion

SCA is a compound mainly derived from the highly toxic drug cantharidin. Although cantharidin has been clinically used for many years, it has been withdrawn from clinical practice in recent years because of its strong side effects (Deng et al., 2017). In recent years, SCA, with its lower side effects and better pharmacological effects, has replaced cantharidin. SCA is currently used for the treatment of various cancers, such as liver cancer, lung cancer, and colon cancer (Wang et al., 2018). However, there are few studies on the therapeutic effect of SCA on breast cancer, and only some clinical data have shown that SCA has a positive effect on the prognosis of breast cancer patients. In addition, our group has confirmed in past studies that SCA can exert anti-cancer effects by regulating the metabolism of breast cancer cells (Pang et al., 2021). In this study, we found that SCA exerts anti-cancer effects by activating autophagy, inhibiting the PI3K–AKT–mTOR pathway, and inducing breast cancer cell apoptosis.

In this study, SCA-treated and control groups received treatment simultaneously, and there was a significant difference in tumor size after 21 days, indicating that SCA can inhibit the growth of tumors in nude mice. In addition, we found that during the treatment process, the body weight of the nude mice in the SCA-treated group showed a slow growth trend, indicating that the nude mice tolerated the SCA treatment well, while the body weight of the nude mice in the model group grew rapidly in the early treatment stage. In the later part of the treatment, the sudden drop in the body weight of nude mice might have been caused by rapid tumor growth. This is consistent with the result that the tumor weight in the SCA-treated group was lower than that in the control group after treatment of nude mice. Furthermore, Ki67 immunohistochemical staining showed that the tumor in the control group was more severe and had great potential for proliferation. Although less toxic than cantharidin, SCA can also cause toxic side effects. Interestingly, the results of hematoxylin-eosin staining showed that SCA treatment did not cause significant organ damage, indicating that the use of SCA within the safe dose range may also be related to insufficient treatment time. It is worth noting that in the *in vitro* experiments, we confirmed that SCA can inhibit the proliferation, invasion, and migration of breast cancer cells, but we did not find a sign of tumor metastasis in nude mice, even in the model group. This may be attributed to the chosen model and the injection location. In general, abundant blood vessel distribution is conducive to tumor metastasis. In addition, the tumor growth time was probably too short to support metastasis.

The cellular regulation of autophagy is a complex process, and at the early stage of autophagy, Beclin1 interacts with PI3K or VPS34, thereby promoting the fusion of autophagosomes with lysosomes. Thus, sufficient expression and interaction of Beclin1 are necessary to execute autophagy (Liang et al., 1999; Morris et al., 2015). p62 is a widely studied autophagy substrate. During autophagosome formation, p62 links LC3 and polyubiquitinated proteins, which are subsequently selectively encapsulated into autophagosomes and ultimately degraded by hydrolases in autophagolysosomes (Wang et al., 2008; Alers et al., 2012). As a hallmark protein, LC3 plays an important role in autophagy. First, LC3-І is modified and processed by ubiquitination and coupled to phosphatidylethanolamine to form LC3-II and localizes to the outer membrane of autophagosomes. Then, LC3-II in the outer membrane is cleaved by Atg4 to produce LC3-І, while LC3-II in the inner membrane is degraded (Wang et al., 2020). Therefore, LC3 is considered a gold indicator of autophagy. Since the discovery of the autophagic process, among a number of signaling pathways, the mTOR signaling pathway, mainly including the PI3K–Akt–mTOR and AMPK–mTOR signaling pathways, is considered to be the most important for the regulation of autophagy (Al-Bari et al., 2020). mTOR is a downstream mediator of the PI3K–Akt pathway, and activated mTORC1 inhibits the autophagic cascade by phosphorylating autophagy protein complexes (Janku et al., 2011). In addition, mTORC2 activation contributes to Akt phosphorylation, and activated AKT can again activate mTOR or directly regulate the FOXO transcription factors to inhibit autophagy (Sun et al., 2005; Liu et al., 2017). In this study, the microarray results suggested that the effect of SCA on breast adenocarcinoma cells might be dependent on the regulation of the PI3K–Akt–mTOR signaling pathway. Indeed, we detected the levels of related proteins in the cells and found that the PI3K–Akt–mTOR signaling pathway was inhibited by SCA. Furthermore, we found that autophagy might also largely contribute to the role of SCA in this process. The results from the autophagy detection assay and TEM also confirmed that SCA induced autophagy in breast cancer cells. Furthermore, the p62 levels were found to be decreased, while the levels of LC3-II and Beclin1 were significantly increased, suggesting an increased level of autophagy in breast cancer cells after SCA treatment.

Autophagy is a double-edged sword; although moderate autophagy can promote the reuse of intracellular materials and inhibit apoptosis, the end result of excessive autophagy is often apoptosis (Ma et al., 2018; Patra et al., 2019). Notably, the activation of apoptosis-associated caspases often leads to the shutdown of the autophagic process. Indeed, in tumor cells, rapid proliferation leads to prolonged exposure to nutrient stress, and intracellular autophagy and apoptosis are simultaneously activated (Marino et al., 2014). In this study, TEM results showed that autophagy was significantly induced (increased autophagosomes) after SCA treatment. Interestingly, while we did not observe a significant enhancement of apoptotic features, caspase3 activity and SCA-induced caspase3 activation were detected. We speculate that under SCA treatment, the PI3K–Akt–mTOR pathway was inhibited in breast cancer cells, and that the low activity of mTOR was not enough to exert an inhibitory effect on autophagy so that the autophagic process could be activated. Meanwhile, the resistance of the PI3K–Akt–mTOR pathway to environmental pressure is turned off, and eventually excessive autophagy induces cell apoptosis. Owing to the short duration of action and the fact that the cells were in the early stage of apoptosis, no obvious apoptotic characteristics were observed.

In summary, this study confirmed that SCA exerts good anti-breast cancer effects *in vitro* and *in vivo*. Specifically, it induced excessive autophagy in breast cancer cells by inhibiting the PI3K–Akt–mTOR signaling pathway, ultimately leading to cell apoptosis. However, the limitation of this study is the absence of data combined with the clinical use of SCA to treat tumors. In fact, SCA as an antitumor agent has been in clinical use for many years. In addition, while we concluded that SCA induces autophagy and apoptosis in breast cancer, which is supported by both *in vitro* and *in vivo* experiments, TEM observations showed that the characteristics of autophagy were more obvious compared with those of apoptosis. Studies have shown that the regulation of apoptosis by autophagy is bifrontal; therefore, the occurrence of apoptosis after SCA treatment may be regulated by autophagy and present an indistinct feature. The specific regulatory mechanism of autophagy on apoptosis still needs further investigation. Nevertheless, our results indicate that SCA is an effective potential drug for the treatment of breast cancer and provide a scientific basis for the clinical application of SCA in breast cancer treatment.

## Data Availability

The datasets presented in this study can be found in online repositories. The names of the repository/repositories and accession number(s) can be found below: NCBI database under accession number PRJNA872001.

## Abbreviations

AMPK, AMP-activated protein kinase; breast cancer, breast cancer; CCK-8, cell counting kit-8; DAPI, 4′,6-diamidino-2-phenylindole; EdU, 5-ethynyl-2′-deoxyuridine; mTOR, mammalian target of rapamycin; PI3K, phosphoinositide 3-kinase; SCA, sodium cantharidate; TEM, transmission electron microscopy.
