# Supplementary figures and images for: Sodium cantharidate promotes autophagy in breast cancer cells by inhibiting the PI3K–Akt–mTOR signaling pathway
Source: Front Pharmacol. 2022 Nov 2;13:1000377. doi: 10.3389/fphar.2022.1000377 (PMC9666387; doi:10.3389/fphar.2022.1000377)

## Slide 1
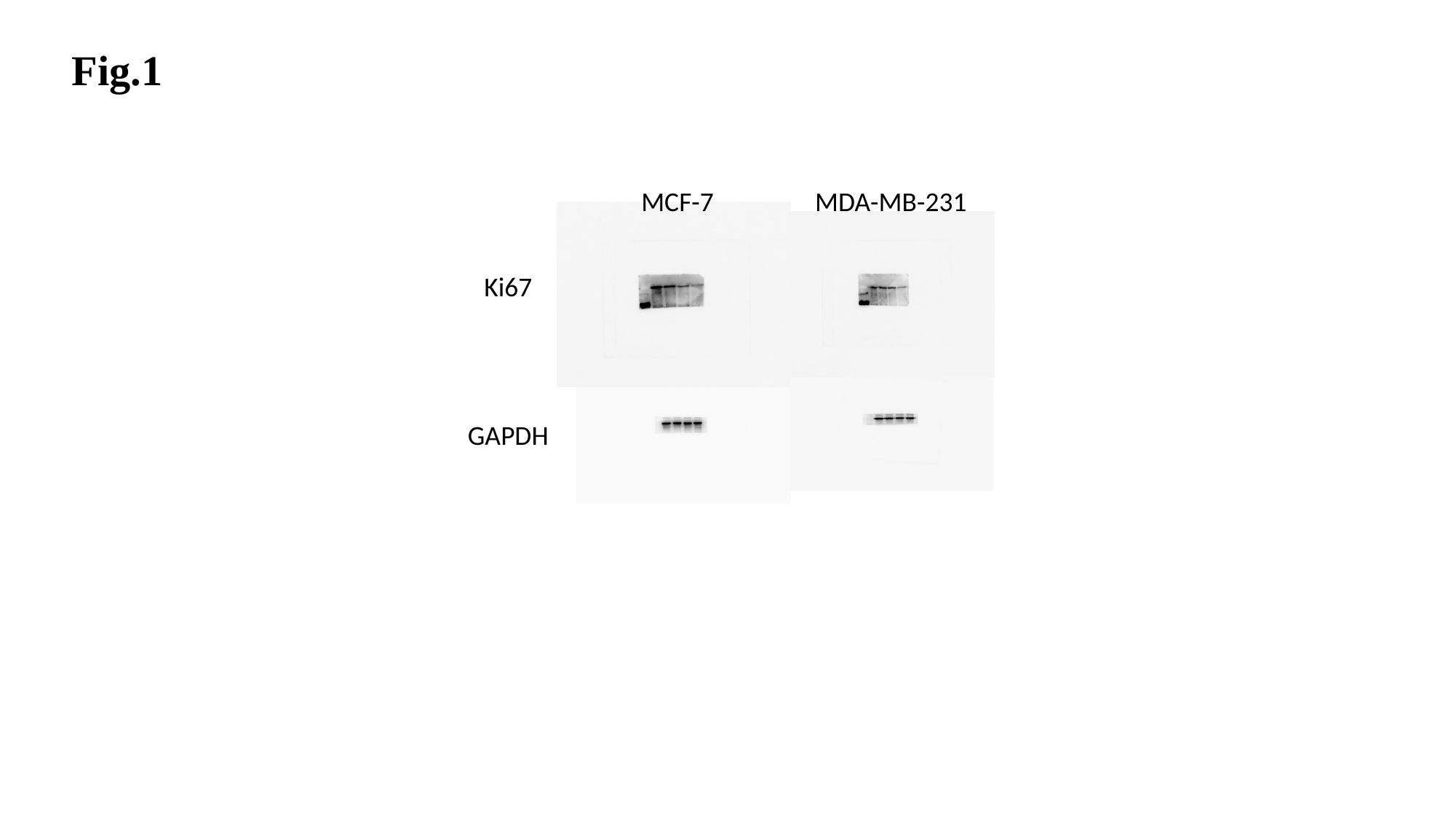

Fig.1
MCF-7
MDA-MB-231
Ki67
GAPDH

Supplement: Supplementary file 3 [file Presentation1.PPTX]

## Slide 1
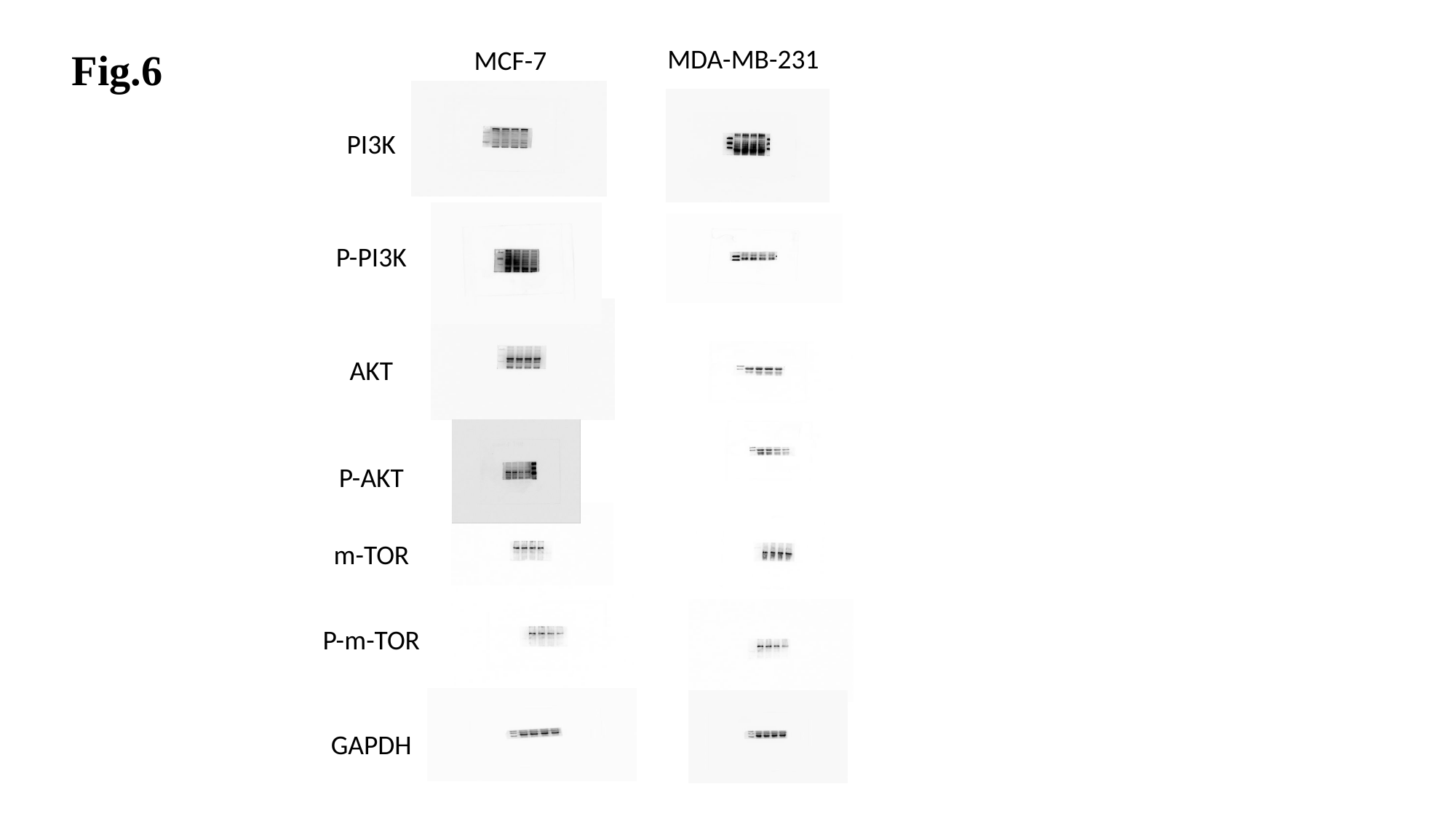

MDA-MB-231
MCF-7
PI3K
P-PI3K
AKT
P-AKT
m-TOR
P-m-TOR
GAPDH
Fig.6

Supplement: Supplementary file 4 [file Presentation4.PPTX]

## Slide 1
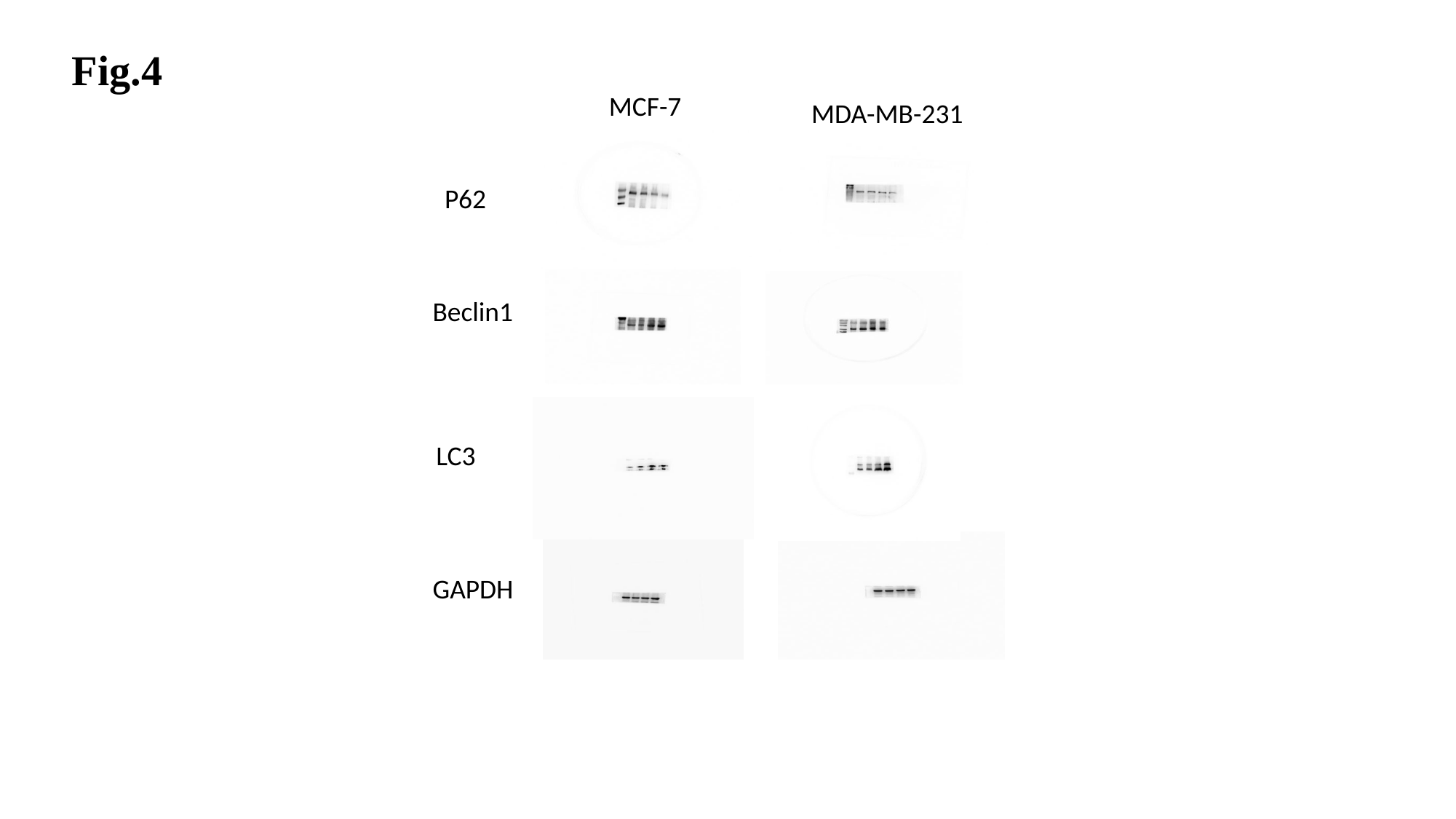

Fig.4
MCF-7
MDA-MB-231
P62
Beclin1
LC3
GAPDH

Supplement: Supplementary file 6 [file Presentation3.PPTX]

## Slide 1
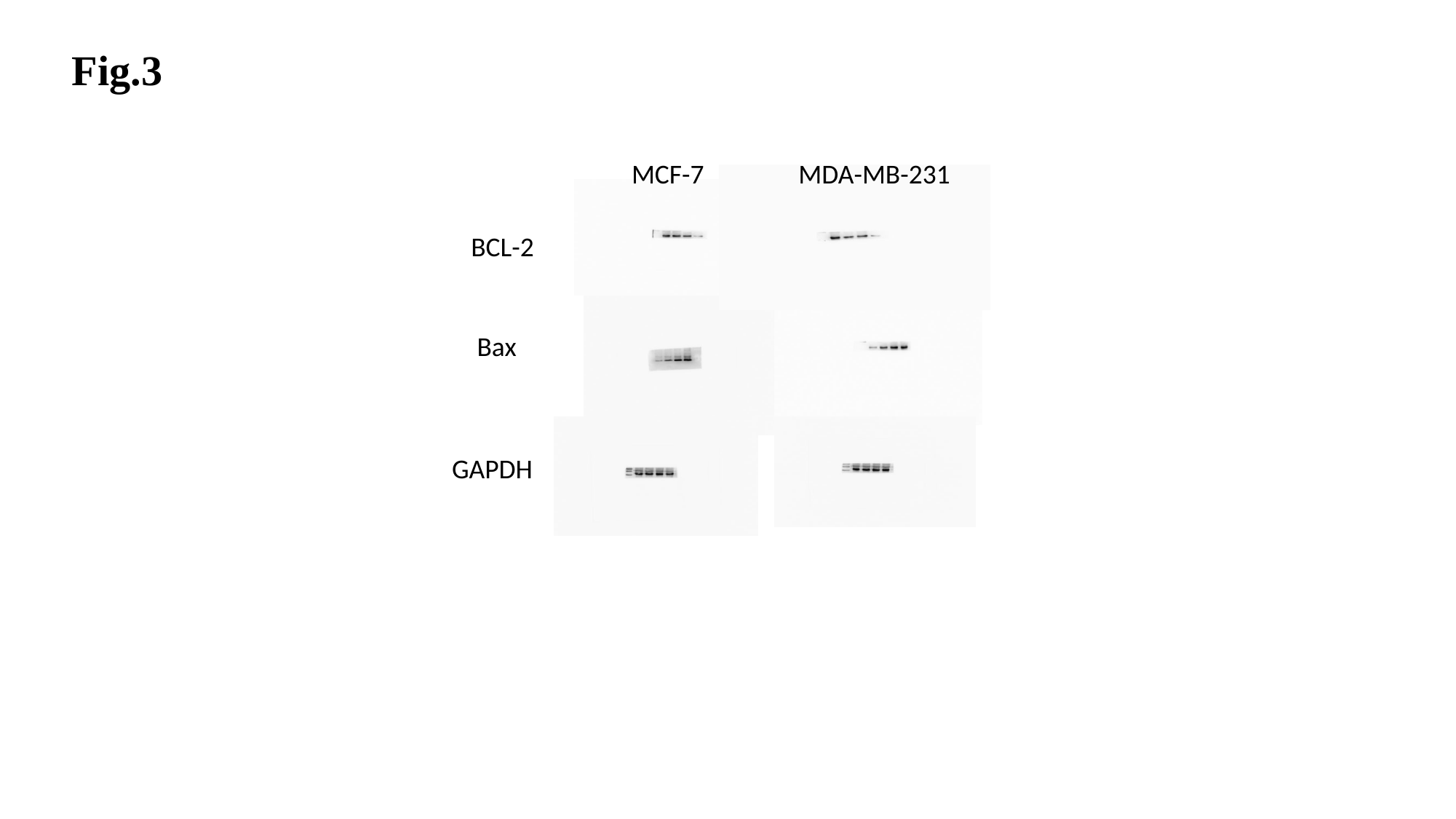

Fig.3
MCF-7
MDA-MB-231
BCL-2
Bax
GAPDH

Supplement: Supplementary file 7 [file Presentation2.PPTX]

## Slide 1
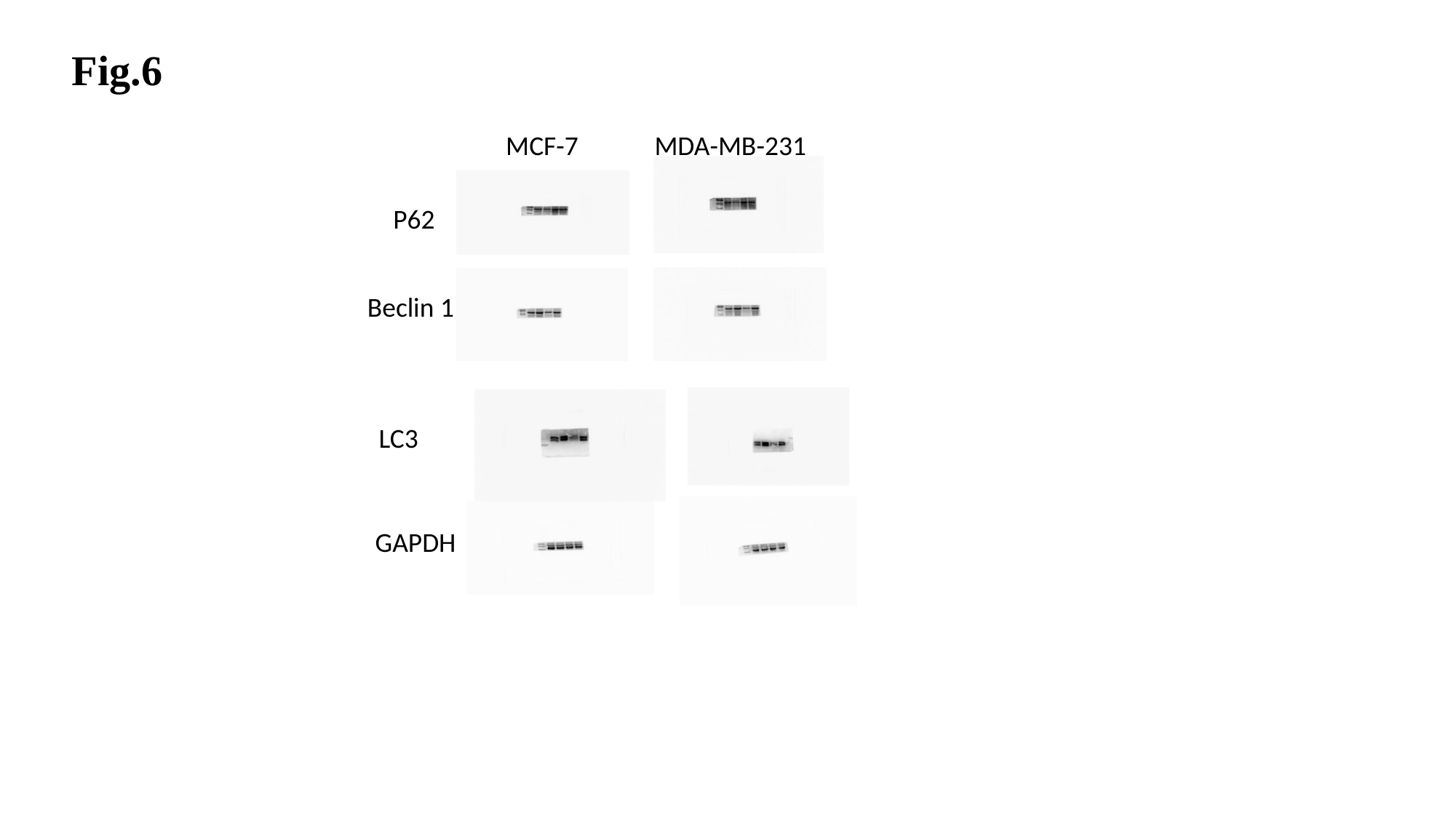

Fig.6
MCF-7
MDA-MB-231
P62
Beclin 1
LC3
GAPDH

Supplement: Supplementary file 8 [file Presentation5.PPTX]
